# Validity of the Wrist-Worn Polar Vantage V2 to Measure Heart Rate and Heart Rate Variability at Rest

**DOI:** 10.3390/s22010137

**Published:** 2021-12-26

**Authors:** Olli-Pekka Nuuttila, Elisa Korhonen, Jari Laukkanen, Heikki Kyröläinen

**Affiliations:** 1 Faculty of Sport and Health Sciences, University of Jyväskylä, 40014 Jyväskylä, Finland; elisa.m.korhonen@student.jyu.fi (E.K.); heikki.kyrolainen@jyu.fi (H.K.); 2 Institute of Public Health and Clinical Nutrition, University of Eastern Finland, 70210 Kuopio, Finland; jari.laukkanen@ksshp.fi; 3 Central Finland Health Care District, Department of Internal Medicine, 40620 Jyväskylä, Finland

**Keywords:** photoplethysmography, heart rate monitor, wearables

## Abstract

Heart rate (HR) and heart rate variability (HRV) can be monitored with wearable devices throughout the day. Resting HRV in particular, reflecting cardiac parasympathetic activity, has been proposed to be a useful marker in the monitoring of health and recovery from training. This study examined the validity of the wrist-based photoplethysmography (PPG) method to measure HR and HRV at rest. Recreationally endurance-trained participants recorded pulse-to-pulse (PP) and RR intervals simultaneously with a PPG-based watch and reference heart rate sensor (HRS) at a laboratory in a supine position (n = 39; 5-min recording) and at home during sleep (n = 29; 4-h recording). In addition, analyses were performed from pooled laboratory data (n = 11344 PP and RR intervals). Differences and correlations were analyzed between the HRS- and PPG-derived HR and LnRMSSD (the natural logarithm of the root mean square of successive differences). A very good agreement was found between pooled PP and RR intervals with a mean bias of 0.17 ms and a correlation coefficient of 0.993 (*p* < 0.001). In the laboratory, HR did not differ between the devices (mean bias 0.0 bpm), but PPG slightly underestimated the nocturnal recordings (mean bias −0.7 bpm, *p* < 0.001). PPG overestimated LnRMSSD both in the laboratory (mean bias 0.20 ms, *p* < 0.001) and nocturnal recordings (mean bias 0.17 ms, *p* < 0.001). However, very strong intraclass correlations in the nocturnal recordings were found between the devices (HR: 0.998, *p* < 0.001; LnRMSSD: 0.931, *p* < 0.001). In conclusion, PPG was able to measure HR and HRV with adequate accuracy in recreational athletes. However, when strict absolute values are of importance, systematic overestimation, which seemed to especially concern participants with low LnRMSSD, should be acknowledged.

## 1. Introduction

Wearable technology has been ranked in the top three fitness trends in the ACSM annual survey for fitness professionals since 2016 [1]. Wearables include fitness trackers, smartwatches, heart rate monitors, and GPS tracking devices [1] that may provide information on functions, such as steps, estimated energy expenditure, and heart rate (HR) [2,3]. It has been suggested that data collected via wearables may be useful in a variety of populations. For example, HR and heart rate variability (HRV) provides information on autonomic nervous system (ANS) regulation, and HRV could be used as an indirect marker of cardiac parasympathetic nervous system activity [4]. Recently, Altini and Plews [5] illustrated how resting HRV may provide additional insights compared to HR only on responses to different types of stressors. Furthermore, monitoring of resting HR [6] or heart rate variability (HRV) [7,8] could be beneficial for predicting the risk of cardiovascular events, such as acute coronary syndromes or strokes. In the training context, daily resting HRV recordings have been used in the endurance training prescription of untrained [9], recreationally trained [10], and well-trained [11] participants, inducing greater improvements in endurance performance compared to predefined training. While there are more wearables available that claim to measure meaningful results, their validity should also be critically studied [2,12] and sources of inaccuracies acknowledged [13,14].

HR monitors have typically demanded a strap for accurate results, but several alternative innovations have been introduced lately. Resting HR and HRV can nowadays be measured with reasonable accuracy from a fingertip via a mobile app [15], from a ring [16], wrist-worn watches [17,18], and sensors placed under a mattress [19]. An additional benefit in terms of feasibility is that many of these devices collect the data automatically during the night [16,18,19], allowing data collection to occur without extra effort compared to traditional morning HR recordings.

Most of the alternative HR, or actual pulse rate, methods are based on photoplethysmography (PPG) [15,16,17,18]. The rationale behind the technology is that when the skin is exposed to LED-emitted light, the change in blood volume can be estimated through the intensity of the reflected light [15,18]. After systole, higher blood volume, and reduced intensity of the reflected light can be observed, while during diastole, blood volume decreases, and the intensity of the reflected light increases [15,18]. Based on these observations, pulse-to-pulse intervals (PP intervals) can be calculated. While pulse rate variability (PRV) may potentially be affected by factors not strictly related to HRV, it could even be argued that PRV should not illustrate exactly similar results compared to HRV [14]. However, PRV and HRV seem to agree quite well at least at resting conditions [20], and previous studies comparing PPG- and electrocardiography-derived HRV during sleep have shown decent agreement between the methods [16,18].

The validity of PPG-based wearables has been previously assessed mainly during exercise [21,22,23,24] and regarding HR only, but the capability of the current method (Polar Precision Prime^TM^) to measure HRV in general, and either HR or HRV during sleep has not yet been examined. Since resting HR, and especially HRV assessments, provide relevant information that could be used to monitor health-related aspects and recovery from training if measured accurately, the purpose of this study was to analyze the validity of the Polar Vantage V2 wrist-based method to measure HR and HRV at rest and during sleep.

## 2. Materials and Methods

### 2.1. Participants

A total of 41 participants were recruited for a larger study project, during which the current validation protocol was executed. Participants were healthy, recreationally endurance-trained, 36 ± 7 year-old males (n = 21) and females (n = 20). Laboratory recordings were successfully conducted by 39 participants. One recording failed due to the early termination of the PPG recording, which was not noticed before analyzing the raw data. In addition, data from one participant were excluded due to poor data quality (more than 20% of the recorded data points missing after the applied proprietary filter). Nocturnal recordings were successfully performed by 29 participants. Data were unavailable due to missing the raw PPG-data (n = 5) or the raw reference data (n = 4), failing of the download process (n = 2), and dropping out from the study before performing the recording (n = 1).

The study protocol was approved by the ethics committee of the University of Jyväskylä.

### 2.2. Experimental Overview

**Figure 1:**
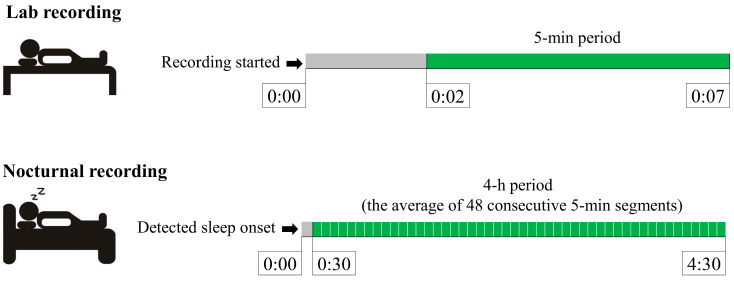
Segments used in the analysis of laboratory and nocturnal recordings.

The validity of the Polar Vantage V2 (Polar Electro Oy, Kempele, Finland) wrist-based PPG-method (PPG) to measure HR and HRV was assessed at rest in two different conditions: (1) Awake in a controlled laboratory setting, and (2) During the night sleep at home (Figure 1). On both occasions, PPG-derived values were compared to the Polar H10 (Polar Electro Oy) heart rate sensor (HRS), which has been reported to be highly accurate in the detection of RR-intervals at rest and during exercise [25]. Average HR and LnRMSSD (the natural logarithm of the root mean square of successive differences) were analyzed in both conditions and from both devices. Concerning variables were used since 4-h average HR and RMSSD are provided by the watch for the actual user in the “Nightly Recharge” feature. In addition, RMSSD [26] and its log-transformed version, LnRMSSD [27], have been suggested to be the most suitable markers for monitoring HRV in the context of training and recovery.

In the laboratory recordings, PPG and HRS were used simultaneously in a supine position during spontaneous breathing. The firmware update, provided by the manufacturer, allowed recording the data with both devices at the same time. The heart rate strap was moistened and attached tightly around the torso at the level of the xiphoid process. The watch was attached to the wrist according to the instructions provided by the manufacturer. The participants were advised to lie at rest without moving during the 7-min data collection, and the last 5-min period was used in the further analysis.

In the nocturnal recordings, PPG and HRS data were collected simultaneously with a watch and strap that was either connected to the Polar sensor logger-application (n = 24) or another Vantage V2 watch (n = 5). The participants were advised to attach the strap and the watch with the same instructions as during the laboratory visit. HRS recording was started manually when the participants went to sleep, while PPG recording started automatically after detected sleep onset. The 4-h analysis period started 30 min after detected sleep onset, in accordance with the “Nightly Recharge” feature in the watch.

### 2.3. HRV Analysis

The test app (for recording raw data) and software (Debugtool for extracting data from the watch; OHR log decoder for opening the packed data) that were specifically provided by the manufacturer for the research purpose allowed the collection and extraction of raw PP intervals from the watch. In the laboratory recordings, PPG-derived PP intervals and HRS-derived RR intervals were exported to Excel. The data were visually inspected to confirm the matching of the data points between the devices. Furthermore, reference data were critically evaluated for possible artifacts. The cardiologist confirmed two physiologically unlikely RR-interval lengths, and respective data points were removed from PPG and HRS to avoid distorting the results. While artifact correction is a crucial part of the HRV analysis, a similar proprietary filter that is used in the “Nightly Recharge” feature was applied for the PPG data to analyze results as they would have been provided by the watch. The exact algorithm behind the filter is not available, but it may remove data points that are estimated to represent insufficient data quality. The percentage of missing data points after applying the filter is reported in the results section. Average HR and LnRMSSD were calculated for PPG and HRS. In addition, similar to the work of Hernando et al. [17], pooled results were used for comparison between the PP and RR intervals.

The same tools were used for the extraction of nocturnal PP intervals, while RR data from HRS were exported via the Polar sensor logger-application or from Polar Flow. Data were first matched based on timestamps, and further synchronized according to the offset-values to induce the best signal fit. The “Nightly Recharge” algorithm, which uses a proprietary filter and averages data to 5-min segments, was applied to the HRS and PPG data after the synchronization. The final analysis period for nocturnal recordings consisted of an average 4-h time period (48 consecutive 5-min segments) starting 30 min after sleep onset. Sleep onset was automatically detected by the watch, and the accuracy of the method has been reported previously [28].

The exact algorithm behind the pulse wave detection of the current PPG method is not published by the manufacturer. However, based on the white paper [29] available on the company’s website, certain aspects regarding the method are possible to clarify. The watch basically calculates the time between high and low light intensities, which varies between systolic and diastolic phases due to changes in the blood volume in the arteries. The watch contains multiple LEDs (a total of 10 in Vantage V2) using several wavelengths of light. All paths provide their own signals, and these can be compared to confirm the origin of the signal (pumping heart, not movement). Another feature that is used to overcome issues related to data quality involves a 3D acceleration sensor that allows differentiating volumetric changes caused by the pumping heart from the changes caused by movements. Based on information combined from these sources, interbeat intervals could be obtained.

### 2.4. Statistical Analysis

All values are expressed as mean and standard deviation (SD). The normal distribution of the data was verified with the Shapiro–Wilk test. To assess differences between HRS- and PPG-derived results, paired-samples *t*-test, mean absolute error (MAE), and mean absolute percentage error (MAPE) were analyzed separately for 5-min and 4-h segments. Relationships between the methods were examined with the Pearson, intraclass (ICC), and Lin’s concordance (CCC) correlation coefficients, and the Bland–Altman plot was used to examine agreement between the HRS and PPG methods. Since pooled PP and RR interval data were not normally distributed, Wilcoxon signed-rank test was used for comparison between methods and Spearman correlation for regression analysis. The statistical significance level was set to *p* < 0.05. Analyses were performed with Microsoft Excel 2010 (Microsoft Corporation, Redmond, WA, USA) and IBM SPSS Statistics v.26-programs (SPSS Inc., Chicago, IL, USA).

## 3. Results

### 3.1. Laboratory Recordings

**Figure 2:**
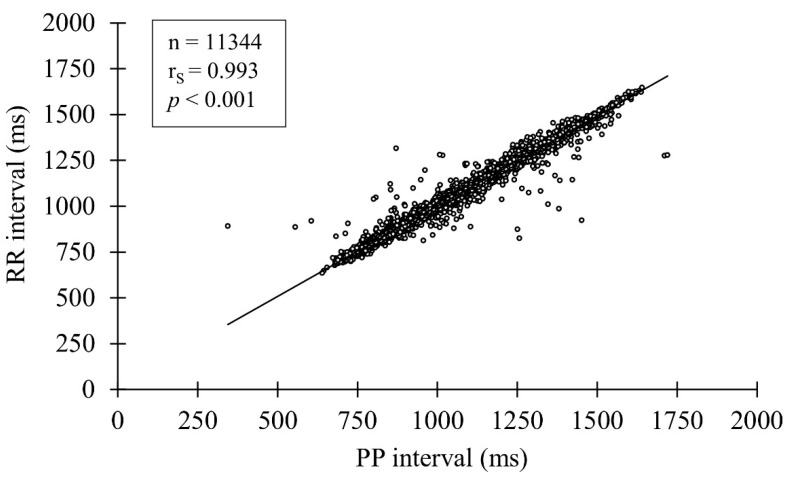
Correlation between the PP and RR intervals.

**Figure 3:**
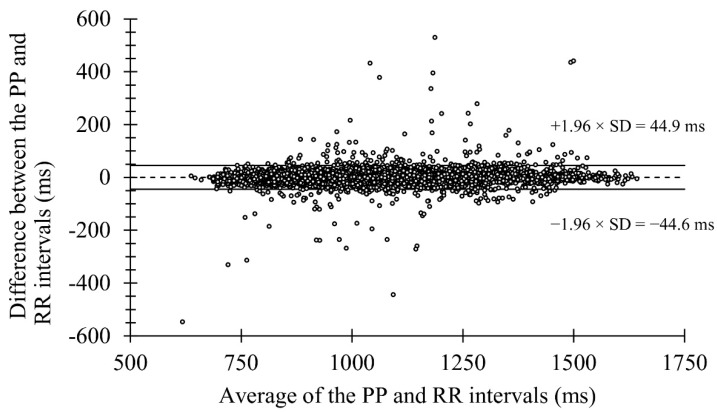
Bland-Altman plot presenting the mean bias and limits of agreement in the laboratory recordings.

No differences were observed between the pooled PPG-derived PP intervals and HRS-derived RR intervals (mean bias 0.2 ± 2.2%) (Table 1). In addition, a very strong correlation (Figure 2) and high agreement (Figure 3) were observed between the methods.

**Figure 4:**
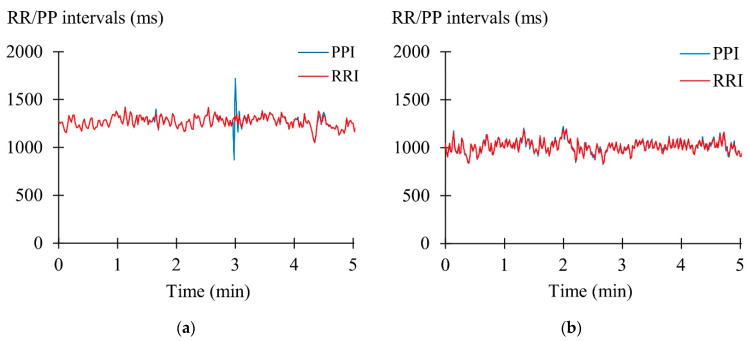
(**a**) Participant with an erroneous extra beat and missed beat, (**b**) Participant with a good agreement between the PP and RR intervals.

When individual 5-min segments were compared, HR did not differ between the methods (mean bias 0.0 ± 0.1%), but LnRMSSD was overestimated (mean bias 5.4 ± 6.3%, *p* < 0.001) by PPG (Table 2). After the Polar proprietary filter was applied to the data, 0.66 ± 1.85% of the data points were excluded. In Figure 4, two case examples are presented, illustrating good agreement and the most typical type of error causing a difference between the measurements.

### 3.2. Nocturnal Recordings

**Figure 5:**
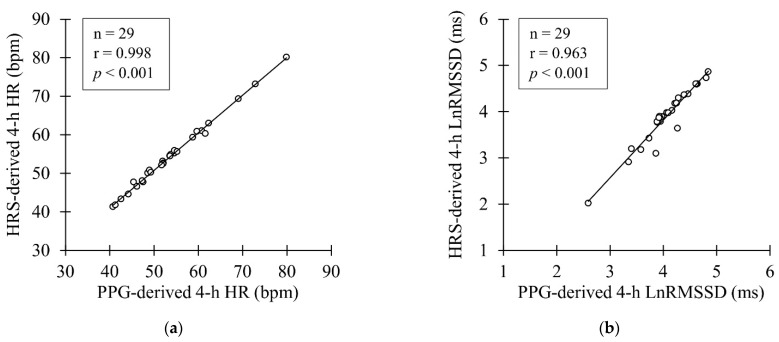
(**a**) Correlation between PPG- and HRS-derived nocturnal HR (**b**) Correlation between PPG and HRS-derived nocturnal LnRMSSD.

In the nocturnal recordings, small but significant underestimation was observed by PPG in HR (mean bias −1.3 ± 1.2%, *p* < 0.001), and overestimation in LnRMSSD (mean bias 5.1 ± 7.3%, *p* < 0.001) (Table 2). However, a very strong correlation was found between the methods in HR and LnRMSSD (Figure 5). After applying the proprietary filter, 0.22 ± 0.85% of the 5-min data points was excluded, equal to three 5-min segments in total.

**Figure 6:**
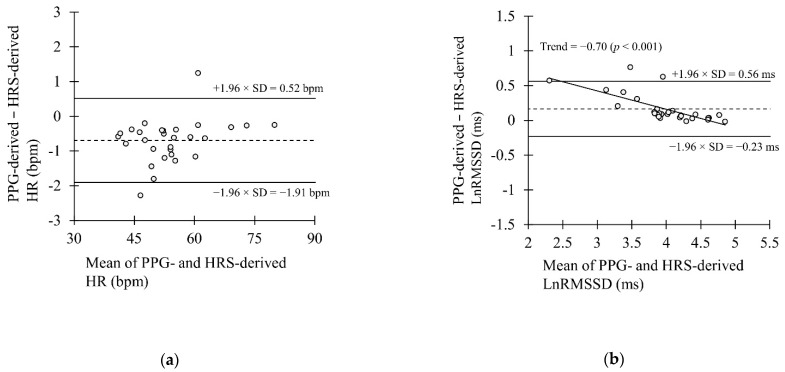
Bland-Altman plot presenting the mean bias and the limits of agreement in the laboratory recordings in the (**a**) nocturnal HR recordings and (**b**) nocturnal LnRMSSD recordings.

Figure 6 illustrates the Bland–Altman plot for nocturnal HR and LnRMSSD. Limits of agreement were defined as mean bias ± 1.96 × SD of differences between PPG and HRS (−0.69 ± 1.21 bpm for HR and 0.17 ± 0.40 ms for LnRMSSD).

## 4. Discussion

The main findings of the study were that the PPG method was able to measure PP intervals in the laboratory conditions with very good accuracy as compared to the HRS-derived RR intervals. In the nocturnal recordings, HR was slightly underestimated (bias −0.7 bpm) and LnRMSSD was overestimated (bias 0.17 ms) by PPG. Based on the Bland–Altman plot, overestimation in the LnRMSSD seemed to especially concern participants with low HRV. Correlation analysis illustrated strong correlations between the devices in both markers. Based on the results, the current PPG method could be regarded as sufficiently accurate to monitor nocturnal HR and HRV in recreational athletes.

While HR and HRV could be monitored with an increasing number of wearables, it is surprising how poorly their validity has been examined in many cases. One certain challenge is that the data given by the wearables (actually measured data vs. developed own metrics), as well as the analysis methods (measurement duration, time of the day), vary quite a lot between manufacturers. In addition, new manufacturers and products are continuously emerging [3], making it challenging to maintain updated research. Recently, Stone et al. [12] compared several PPG-based methods in the assessment of resting HR and RMSSD. In HR, MAPE compared to reference-ECG varied between 1.2% and 17.3%, while for RMSSD it varied between 4.1% and 112.4%. Compared to those results, current errors were smaller or at the lower end of the spectrum. However, none of the applied methods were wrist-based, and when setting current results into the perspective of wrist-based wearables somewhat comparable validation studies have been performed using Whoop’s wrist-strap during slow-wave-sleep [18] and the Apple Watch during relax and stress situations [17]. Bellenger et al. [18] found that Whoop’s PPG method accurately measured HR (bias ≤ 0.39%), but a larger error was observed between the devices in LnRMSSD (bias ≥ 1.66%). In turn, Hernando et al. [18] found no difference between the reference device and Apple Watch in the HR or RMSSD during relaxation. Possible explanations for the lower error values compared to the current study may relate to the different time-period used in the nocturnal analysis [18] and filters applied to the artifact correction [17,18]. It should also be acknowledged that Bellenger et al. [18] had only six participants, which makes it hard to draw broader conclusions, as in the present study it was observed that the agreement between devices may also vary between participants. Furthermore, Hernando et al. [17] reported only pooled results, leaving individual results and between-individual variability in the accuracy speculative.

In the ICC analysis, it has been suggested that values above 0.90 indicate excellent reliability [30]. The current PPG method fulfilled this criterion in the lab and sleep recordings of HR and LnRMSSD. On the other hand, in CCC, which is suggested to illustrate concordance between methods better than other correlation coefficients [20], values were slightly lower and below 0.90 in LnRMSSD, while in HR almost perfect relationships were found (r > 0.99) despite the correlation method being used. In previous studies measuring HR and HRV by PPG during the night, comparable ICC values have been observed [18,31] in HR, but in LnRMSSD higher values have also been found [18]. Some studies have reported only linear correlation values, and they have been both slightly higher using the PPG-based method [16] and lower with the ballistocardiography-based method [19] as compared to the present results. Regarding MAE and MAPE, desirable values depend highly on the context and the marker being used, and exact target values are therefore hard to define. Bellenger et al. [18] suggested that the accuracy of the wearables should be examined in the light of the smallest worthwhile change (SWC) of the parameter. In the HR and vagally mediated HRV parameters, Buchheit [26] proposed SWC of ~2 and ~3%, respectively. In the present study, MAPE of HR was lower than this, but LnRMSSD exceeded the value. However, it should be noticed that in 19 out of 29 participants, MAPE was below 3%, and few participants with poor agreement significantly affected the mean results. In addition, Plews et al. [32] have reported that recreational athletes may have higher day-to-day variation as compared to well-trained athletes (CV 10.1% vs. 6.8%), also increasing SWC. In the studies where HRV results have been used in the training prescription, SWC have varied between 1 × SD [9] and 0.5 × SD [11] of the preceding 10–28-day results. However, it is clear that for recovery-monitoring purposes, only sufficiently accurate methods should be used. If the method itself is the most significant source of error affecting the within-day variability, it makes it excessively challenging to find meaningful changes.

When thinking about the inaccuracies in wrist-worn wearables, Bent et al. [13] listed skin type, motion artifacts, and signal crossover as possible sources of error. Since in the current study, participants measured HR and HRV in a supine position, motion artifacts or signal crossover would be expected to be negligible. Although movement artifacts are not a similar problem in the resting measurements as they might be during exercise, movements during sleep may be a slight issue in restless sleepers. The current PPG recording started 0.5 h after detected sleep onset and continued for 4 h in line with protocols used in previous studies [33,34]. Since the first hours of sleep typically have the highest proportion of slow-wave sleep, representing the most restful and stable period of the night [35], the 4-h analysis period may speculatively have some benefits compared to the whole night recordings in terms of data quality. Another aspect possibly affecting the measurement accuracy of the nocturnal recordings in the current study is that they were performed at home without supervision, thus attachment of the watch (placement, tightness) was not strictly controlled. However, participants were instructed in detail during the laboratory measurement on how to wear the watch correctly. The current setting also presented a natural user environment, making the assessment more realistic compared to the laboratory setting. Because, slightly surprisingly, MAPE was even smaller and ICC and CCC were higher in the nocturnal recordings as compared to the controlled laboratory settings, it is unlikely that improper attachment of the watch would have affected the results, in general.

While precise determination of the PP intervals is critical for accurate HR and HRV results, artifact correction and treatment/filtering of the data once abnormal intervals have been found also play a crucial role, especially in the HRV recordings [36]. Detection of abnormal interbeat intervals is typically based on differences between consecutive or multiple previous RR/PP intervals [15,16]. If the measured RR/PP interval differs from the reference value more than a particular threshold, it would be corrected or excluded from the data. This represents a challenge when over-correcting should be avoided but false data points should still be excluded, distorting the results. In the present study, the proprietary filter applied by the watch was more permissible compared to previous studies reporting the amount of missing data being as high as ~10% [17] while resting awake (in the current study ~0.7%) or ~30% [19] during sleep (in the current study ~0.2%). As illustrated in Figure 4, a too permissible filter may ignore quite clear artifacts and may be one of the major reasons behind the inaccuracies. Since most of the manufacturers will not allow consumers to access the raw data in the PPG recordings, these aspects are in most cases hard to examine in detail.

After considering possible sources of inaccuracies in PPG, it is also important to acknowledge that differences in the results may not be related only to possible sources of errors, but also to different method variables are being produced. While traditional HRV, which can be obtained, e.g., via ECG or HR strap, reflects the variation in the RR-intervals that are detected based on the changes in the electrical polarity of the heart [25], PPG-based HRV, or basically PRV, is based on measured variability in the pulse-waves [20]. Yuda et al. [14] listed several transformation phases that may contribute to the potential differences in HRV and PRV: cardiac contraction after R wave causes pressure impulse in the aorta, leading to pulse wave conduction through the arterial wall, and upon reaching the target site, causes changes in blood volume that are finally detected by PPG. Because the aforementioned steps could be affected, e.g., by respiration and blood pressure, the same authors even suggested that HRV and PRV should be taken as separate biomarkers of the ANS function [14]. Schäfer and Vagedes [20] proposed that the relationship between these two methods may be altered especially during physical or mental stress, and interestingly PRV responses may also be affected by the location where the PPG signal is being recorded [37]. Regardless, as has been observed in previous studies examining PPG- and ECG-derived HRV at rest, only minor differences were observed [16,18,31], having hardly any significant effect on the interpretation of the results in settings comparable to the present study.

Current comparisons were performed with a heart rate sensor as a reference instead of a golden standard electrocardiography due to practical reasons. However, the H10 sensor has been examined to be very accurate in the detection of RR-intervals, and previous generation sensors from the same manufacturer (H7) have also been used as a reference in previous studies. The high number of failed recordings decreased the number of participants in the nocturnal measurements. Nevertheless, the current number of participants was most likely sufficient to study the accuracy of the method in the target population of recreationally trained athletes.

## 5. Conclusions

In conclusion, the current PPG method seems accurate in the measurement of PP intervals. In addition, despite nocturnal HR being slightly underestimated by PPG, an almost perfect relationship was observed between the methods. LnRMSSD was overestimated by PPG in the laboratory and nocturnal recordings, and more variation was observed between participants in MAE and MAPE as compared to HR. Overestimation seemed to especially concern participants with low HRV, suggesting that further validation may be recommended for such populations. However, current accuracy could be regarded as sufficient in athletic and healthy populations for the long-term monitoring of HR and HRV, provided that results are interpreted appropriately.

## Figures and Tables

**Table 1:** Comparison between the mean (±SD) pooled PPG-derived PP-intervals and HRS-derived RR-intervals.

	Pooled 5-min Results (n = 11344)
PPG, PP interval (ms)	1022.9 ± 181.1
HRS, RR interval (ms)	1022.7 ± 179.8
Bias (ms)	0.2 ± 22.8
MAE (ms)	10.7 ± 20.2
MAPE (%)	1.1 ± 2.0

PPG, photoplethysmography; HRS, heart rate sensor; MAE, mean absolute error, MAPE, mean absolute percentage error.

**Table 2:** Comparison between the mean (±SD) PPG and HRS in the laboratory (5-min segment) and nocturnal (4-h segment) recordings.

	PPG Mean	HRS Mean	Bias	MAE	MAPE	ICC	CCC
5-min segment (n = 39)							
HR (bpm)	58.6 ± 9.5	58.6 ± 9.5	0.0 ± 0.1	0.0 ± 0.1	0.04 ± 0.08	1.000 ***	1.000
LnRMSSD (ms)	4.01 ± 0.48	3.82 ± 0.51	0.19 ± 0.21 ***	0.20 ± 0.20	5.57 ± 6.14	0.913 ***	0.849
4-h segment (n = 29)							
HR (bpm)	53.8 ± 9.2	54.5 ± 9.0	−0.7 ± 0.6 ***	0.8 ± 0.5	1.49 ± 1.01	0.998 ***	0.995
LnRMSSD (ms)	4.06 ± 0.47	3.90 ± 0.61	0.17 ± 0.20 ***	0.17 ± 0.20	5.23 ± 7.36	0.931 ***	0.890

PPG, photoplethysmography; HRS, heart rate sensor; MAE, mean absolute error, MAPE, mean absolute percentage error; ICC, intraclass correlation coefficient; CCC, Lin’s concordance correlation coefficient. *** *p* < 0.001.

## Data Availability

Data are available from the corresponding author on reasonable request.
